# Transcriptome Sequencing Analysis Reveals a Difference in Monoterpene Biosynthesis between Scented *Lilium* ‘Siberia’ and Unscented *Lilium* ‘Novano’

**DOI:** 10.3389/fpls.2017.01351

**Published:** 2017-08-04

**Authors:** Zenghui Hu, Biao Tang, Qi Wu, Jian Zheng, Pingsheng Leng, Kezhong Zhang

**Affiliations:** ^1^College of Landscape Architecture, Beijing University of Agriculture Beijing, China; ^2^Beijing Collaborative Innovation Center for Eco-environmental Improvement with Forestry and Fruit Trees Beijing, China

**Keywords:** *Lilium*, floral scent, monoterpene, release amount, RNA-Seq

## Abstract

*Lilium* is a world famous fragrant bulb flower with high ornamental and economic values, and significant differences in fragrance are found among different *Lilium* genotypes. In order to explore the mechanism underlying the different fragrances, the floral scents of *Lilium* ‘Sibeia’, with a strong fragrance, and *Lilium* ‘Novano’, with a very faint fragrance, were collected *in vivo* using a dynamic headspace technique. These scents were identified using automated thermal desorption—gas chromatography/mass spectrometry (ATD-GC/MS) at different flowering stages. We used RNA-Seq technique to determine the petal transcriptome at the full-bloom stage and analyzed differentially expressed genes (DEGs) to investigate the molecular mechanism of floral scent biosynthesis. The results showed that a significantly higher amount of *Lilium* ‘Siberia’ floral scent was released compared with *Lilium* ‘Novano’. Moreover, monoterpenes played a dominant role in the floral scent of *Lilium* ‘Siberia’; therefore, it is believed that the different emissions of monoterpenes mainly contributed to the difference in the floral scent between the two *Lilium* genotypes. Transcriptome sequencing analysis indicated that ~29.24 Gb of raw data were generated and assembled into 124,233 unigenes, of which 35,749 unigenes were annotated. Through a comparison of gene expression between these two *Lilium* genotypes, 6,496 DEGs were identified. The genes in the terpenoid backbone biosynthesis pathway showed significantly different expression levels. The gene expressions of 1-deoxy-D-xylulose 5-phosphate synthase (DXS), 1-deoxy-D-xylulose-5-phosphate reductoisomerase (DXR), 4-hydroxy-3-methylbut-2-enyl diphosphate synthase (HDS), 4-hydroxy-3-methylbut-2-enyl diphosphate reductase (HDR), isopentenyl diphosphate isomerase (IDI), and geranyl diphosphate synthase (GPS/GGPS), were upregulated in *Lilium* ‘Siberia’ compared to *Lilium* ‘Novano’, and two monoterpene synthase genes, ocimene synthase gene (*OCS*) and myrcene synthase gene (*MYS*), were also expressed at higher levels in the tepals of *Lilium* ‘Siberia’, which was consistent with the monoterpene release amounts. We demonstrated that the high activation levels of the pathways contributed to monoterpene biosynthesis in *Lilium* ‘Siberia’ resulting in high accumulations and emissions of monoterpenes, which led to the difference in fragrance between these two *Lilium* genotypes.

## Introduction

Floral scent is an important component part of plant volatile compounds. It has been reported that floral scent plays a key role in floral evolution in flowering plants, whose principal function is to attract the pollinators (Galliot et al., 2006; Knudsen et al., 2006). Floral scent contributes to the defense against biotic and abiotic stresses, which is shown to have toxic or deterrent activity against microbes and herbivores, and to ameliorate high temperatures and reduce damage caused by oxidative stress (Knudsen et al., 2006). Floral scent also represents a decisive communication channel between plants and animals (De Vega et al., 2014). Moreover, for ornamental flowers, floral scent is believed to be an important characteristic to evaluate flowers. Consequently, an increasing number of studies have focused on floral scent in recent years (Grausgruber-Grögera et al., 2012; Zhao et al., 2012; Demissie et al., 2013; Sharkey et al., 2013; Feng et al., 2014; Sun et al., 2015; Hattan et al., 2016; Kong et al., 2017).

Floral scents are almost a complex mixture of small volatile molecules with low vapor pressure and are dominated by terpenoids, phenylpropanoids, benzenoid compounds, and fatty acid derivatives (Dudareva and Pichersky, 2000). Terpenoids are the largest class of plant volatiles and represent the most diverse class of chemicals among the myriad compounds (Gershenzon and Kreis, 1999; Tholl, 2015). Terpenoids are biosynthezed through the 2-C-methyl-d-erythritol-4-phosphate (MEP) pathway that mainly mediates the production of monoterpenes in the plastid, and through the mevalonate (MVA) pathway that mainly contributes to the formation of sesquiterpene in the cytosol (Tholl, 2015). The MVA pathway in plants starts with the Claisen-type condensation of two molecules, i.e., acetyl-CoA to acetoacetyl-CoA catalyzed by acetoacetyl-CoA thiolase (AACT). Then, under the catalyzation of HMG-CoA synthase (HMGS), HMG-CoA reductase (HMGR), mevalonate kinase (MK), phosphomevalonate kinase (PMK), and mevalonate diphosphate decarboxylase (MVD) in sequence, acetoacetyl-CoA is converted into isopentenyl diphosphate (IPP), a five-carbon building unit of terpenoid, which is converted into DMAPP through the activity of an IPP isomerase (IDI). Farnesyl diphosphate synthase (FPS) catalyzes the formation of farnesyl diphosphate (FPP), which is converted to sesquiterpene through the catalyzation of terpene synthase (TPS). In the first reaction of the MEP pathway, 1-deoxy-D-xylulose 5-phosphate (DXP) is formed by DXP synthase (DXS) from hydroxyethylthiamine diphosphate (HTD), which is derived from pyruvate and glyceraldehyde-3-phosphate (GAP) in a transketolase-like condensation reaction. Then, through the catalyzation of 1-deoxy-D-xylulose-5-phosphate reductoisomerase (DXR), 4-diphosphocytidyl-2-C-methyl-D-erythritol synthase (MCT), 4-diphosphocytidyl-2-C-methyl-D-erythritol kinase (CMK), 2-C-methyl-D-erythritol 2,4-cyclodiphosphate synthase (MDS), 4-hydroxy-3-methylbut-2-enyl diphosphate synthase (HDS), and 4-hydroxy-3-methylbut-2-enyl diphosphate reductase (HDR) in sequence, a mixture of IPP and DMAPP with a ratio of 5 to 6:1 is produced (Rohdich et al., 2002). In the next step, geranyl diphosphate (GPP) is synthesized from IPP and DMAPP through the activity of GPP synthase (GPS, or GPPS in some studies), which acts as the precursor for the biosynthesis of monoterpenes through the catalyzation of TPS. These two pathways are connected with IPP, which can move between the cytosol and plastid (Dudareva et al., 2005). The genes of enzymes in the MVA and MEP pathways, especially those with important regulatory roles, obtain great attention (Tholl, 2015).

Floral scents vary widely among species in terms of the constituents, number, and relative amounts, which give the flowers their characteristic fragrances (Knudsen et al., 2006). Kishimoto et al. (2011) selected 11 *Dianthus* species and found that the main floral components detected were significantly different among diverse groups divided according to their sensory characteristics. We measured the fragrance composition of 6 tree peony genotypes, and found that the floral scents were qualitatively and quantitatively distinct (Zhao et al., 2012). In a study of floral scents emitted from different *Alstroemeria* genotypes, the scented *Alstroemeria* emits a large amount of terpenes, but these compounds are not detected in the volatiles of unscented *Alstroemeria* (Aros et al., 2012). In recent years, due to relationship with human health, the study of synthesis and emission of plant floral scent becomes a popular field. However, in the breeding research of flowers, the genetic improvement of floral scent falls behind flower type and color, which mainly results from a lack of understanding of the mechanisms in floral scent synthesis.

*Lilium* is a world famous fragrant bulb flower with high ornamental and economic values. *Lilium* occupies a very important position in the global fresh cut flower market and is also used as a common landscaping flower plant in gardens. The production value of lilies is ranked third in the global flower industry. At present, there are more than 100 genotypes found in the market, and new genotypes are continuously being cultivated. However, because the regulation of floral scent has been ignored in *Lilium* breeding, significant differences in fragrance are found among different *Lilium* genotypes. The fragrance of some lilies is too strong, such as the Oriental hybrid lilies, and many people dislike the strong odor. By contrast, some lilies emit a very faint fragrance, such as the Asiatic hybrid lilies. Therefore, genetic improvement is urgently needed with respect to lily floral scent. In our previous study, the difference in fragrance is believed to result from differences in the composition and amount of floral scent released (Zhang et al., 2013). However, little is known about the mechanism underlying differences in the emission of floral scent among different *Lilium* genotypes.

In recent years, the development of new sequencing techniques and *de novo* assembly provides an unprecedented opportunity for non-model species in genome-wide studies (Li et al., 2013; Fan et al., 2015). High-throughput RNA-sequencing (RNA-Seq) has become the most powerful and popular tool to reveal the molecular mechanism of plants, especially for those of which reference genome information is lacking. Identification of differentially expressed genes (DEGs) between different treatments has become a powerful approach to comprehend the complexity of gene regulatory networks (Fan et al., 2015). In addition, through systemic biology investigations, such as Gene Ontology (GO) analysis, and gene family analysis, and gene co-expression networks, our knowledge of gene functions through interrogation of high-throughput transcriptome data has greatly improved. Due to low cost, high efficiency, high accuracy, and sensitive profiles, RNA-Seq has been applied to *Lilium*. Using this approach, the cold response and signaling pathways in *L. lancifolium* (Wang et al., 2014), the key candidate genes in response to vernalization of Oriental lily (Li et al., 2016), and the influence of paclobutrazol on the leaf growth of the *Lilium* Longiflorum-Asiatic hybrid (Zhu et al., 2016) have been analyzed successfully. Therefore, RNA-Seq is an effective approach to explore the biosynthesis mechanism of floral scent in *Lilium*.

In this study, we selected *Lilium* ‘Siberia’, a typical Oriental hybrid lily with a strong fragrance, and *Lilium* ‘Novano’, a typical Oriental hybrid lily with a very faint fragrance as plant materials. The flowers of these two *Lilium* genotypes are white. Since the floral scent of *Lilium* ‘Siberia’ has been analyzed in our previous study (Hu et al., 2016), only the floral scent of *Lilium* ‘Novano’ was collected *in vivo* using a dynamic headspace technique and was identified using automated thermal desorption—gas chromatography/mass spectrometry (ATD-GC/MS), and then the difference was compared. We used RNA-Seq technique to detect the petal transcriptome and analyzed the DEGs. Subsequently, the DEGs associated with floral scent were examined during flower development. The results contribute to our understanding of the mechanism underlying differences of floral scent among *Lilium* genotypes.

## Materials and methods

### Plant materials

In this study, two *Lilium* genotypes, *Lilium* ‘Siberia’ and *Lilium* ‘Novano’ were used as plant materials. The bulbs were cultured in plastic pots (20 cm diameter, 20 cm height) containing medium comprised of peat and vermiculite at a ratio of 2 to 1 in the greenhouse at the Science Park of Beijing University of Agriculture under a 16/8-h light/dark 25/20°C cycle (Hu et al., 2016). The plants were irrigated every 3 days and were supplied with full-strength Hoagland's nutrient solution every 2 weeks (Hu et al., 2016). The flowering period was divided into 4 stages, i.e., the bud stage (BS), half-bloom stage (HS), full-bloom stage (FS), and late-bloom stage (LS). The floral scents were collected and analyzed during these 4 stages. Petals were also collected during these 4 stages and were immediately frozen in liquid nitrogen prior to further analysis. The FS petals were used to detect the transcriptome. Three biological replicates were collected per stage.

### Floral scent collection and analysis

Using dynamic headspace sampling the floral scent was collect (Hu et al., 2016). An individual flower was placed in a Reynolds oven bag (16 × 17.5 in), which releases and absorbs few volatiles. A stainless steel tube (0.25 × 3.5 in) containing Tenax-GR (60–80 mesh, Chrompack) was used as the volatile trap, and care was taken to ensure the flower was not touched (Hu et al., 2016). A portable air sampler (QC-1; Beijing Municipal Institute of Labor Protection, China) served as the pump, and air filtered by charcoal was pumped into the bag. The volatiles were collected for 20 min at a flow rate of 300 mL·min^−1^ (Hu et al., 2015, 2016). Then, the stainless steel tubes were sealed and placed in a refrigerator.

The automated thermal desorption—gas chromatography/mass spectrometry (ATD-GC/MS) technique was used to analyze the floral scent. The floral scent collected in the stainless steel tube was desorbed by heating in an ATD (Auto Thermal Desorber, TurboMatrix 650, PerkinElmer) at 260°C for 10 min and was then cryofocused in a cold trap where the temperature was maintained at −25°C for 3 min. Subsequently, the cold trap was rapidly heated to 300°C, which was maintained for 5 min, to transport the volatiles to a GC (Clarus 600, Perkin Elmer). The GC was equipped with a capillary DB-5MS column (30 m × 0.25 mm i.d. with a 0.25-μm film thickness). Helium was used as the carrier gas. The GC was programmed at 40°C for 2 min, 4°C·min^−1^ up to 160°C, followed by 20°C·min^−1^ up to 270°C, and held at 270°C for 3 min. The MS (Clarus 600T, Perkin Elmer) was operated in EI ionization mode at 70 eV, and a mass scan range of 29–600 amu was monitored. The interface and ion source temperatures were 250°C and 220°C, respectively.

According to the retention indices, the compounds were identified by searching the NIST08 and WILEY library in the TurboMass Ver5.4.2 software. α-pinene (Fluka, USA) was used as an external standard to determined the release amounts of volatile components by dissolution in ethyl acetate with different solution concentrations (Hu et al., 2009, 2015, 2016), and μg·h^−1^ was used as the unit.

### RNA extraction

Total RNA was extracted from the lily petals at FS using RNAiso Plus (TransGen Biotech, Beijing, China). The quality and quantity of purified RNA were examined using a NanoDrop ND-1000 UV/Visible spectrophotometer (Wilmington, DE, USA). The RIN (RNA integrity number) values (>8.0) of these samples were assessed using an Agilent 2100 Bioanalyzer (Agilent Technologies, Santa Clara, CA, USA) for gel electrophoresis. High-quality RNA was used in cDNA library construction and Illumina deep sequencing.

### Construction of cDNA library for illumina sequencing

The construction of the libraries and the RNA-Seq were performed by the Biomarker Biotechnology Corporation (Beijing, China). mRNA was enriched and purified with oligo(dT)-rich magnetic beads and then broken into short fragments. Using these cleaved mRNA fragments as templates, first- and second-strand cDNA were synthesized. The resulting cDNAs were then subjected to end-repair and phosphorylation using T4 DNA polymerase and Klenow DNA polymerase. Subsequently, an ‘A’ base was inserted as an overhang at the 3′ ends of the repaired cDNA fragments, and Illumina paired-end solexa adaptors were subsequently ligated to these cDNA fragments to distinguish the different sequencing samples. The products of the ligation reaction were purified and selected on 2% agarose gel for downstream enrichment. Then the purified cDNA template was enriched by PCR amplification. Finally, 6 libraries were sequenced using an Illumina HiSeq™ 2500 (SRA accession number: SRP112400). Data analysis and base calling were performed using the Illumina instrument software.

### *De novo* assembly and annotation

The raw reads were first filtered to identify clean reads by removing the reads with only adaptor sequences and unknown nucleotides >5%, as well as low quality reads. The clean reads were then assembled *de novo* using the Trinity platform based on the parameters “K-mer = 25, group pairs distance = 300” (Li et al., 2016). The short reads were first assembled into longer contigs on the basis of their overlapping regions. Then, different contigs were further recognized by mapping clean reads back to the corresponding contigs based on their paired-end information, and then the gene sequences were obtained. Finally, the potential gene sequences were clustered to gain unigenes using the TGI tool (Pertea et al., 2003). In order to annotate, the unigene sequences were searched against a series of protein databases using BLASTx, with a cut-off *E*-value of 10^−5^, including the NCBI Cluster of Orthologous Groups of proteins (COG), the Gene Ontology (GO), the Kyoto Encyclopedia of Genes and Genomes (KEGG), the NCBI eukaryotic Orthologous Groups (KOG), the Swiss-Prot, NCBI non-redundant (Nr), and the Pfam. The deduced amino acid sequences of unitranscripts were demanded to be longer than 70% of the corresponding sequences. If a unigene met the criteria, it was assumed to contain a near full-length contig. Alternatively, targeted assembly was performed to obtain even greater coverage of the respective genes. All reads in the databases examined were mapped to the reference sequences, and the mapped reads were then assembled using clustering and CAP3.

### Expression annotation

To evaluate the depth of coverage, all usable reads were realigned to each unigene using SOAPaligner and then normalized into RPKM values (reads per kb per million reads). The unigenes showed differential expression levels among different samples were calculated based on the ratio of the RPKM values. In order to compute the significance of the difference in transcript abundance, the threshold of the *P*-value in multiple tests was idenfied using the false discovery rate (FDR) control method. Only the unitranscripts with an absolute value of log2 ratio ≥2 and an FDR significance score <0.001 were used for subsequent analysis. If FDR was lower than 0.05 and the highest RPKM (reads per kilobase per million reads) of the unigene was twice that of the lowest one, the unigene was considered as differentially expressed gene (Zheng et al., 2015).

### Quantitative real-time PCR (qRT-PCR) validation

The unigenes were subjected to quantitative real-time PCR (qRT-PCR) with specific primers (Supplementary Table 1). SYBR Green PCR Master Mix was used to detect the PCR products on a 7900 HT Sequence Detection System (Applied Biosystems). QRT-PCR was performed using the SYBR Premix Ex Taq Kit (TaKaRa) according to the manufacturer's protocol. The results were normalized to the expression level of the constitutive actin gene, and a comparative Ct method (2^−ΔΔct^) of relative quantification was used to evaluate the quantitative variation (Zheng et al., 2015). There were three independent biological replicates and three technical replicates of each biological replicate for each sample. The samples in qRT-PCR validation were the same as those for Illumina sequencing.

## Results

### The floral scent analysis of *Lilium* ‘Siberia’ and *Lilium* ‘Novano’

**Figure 1:**
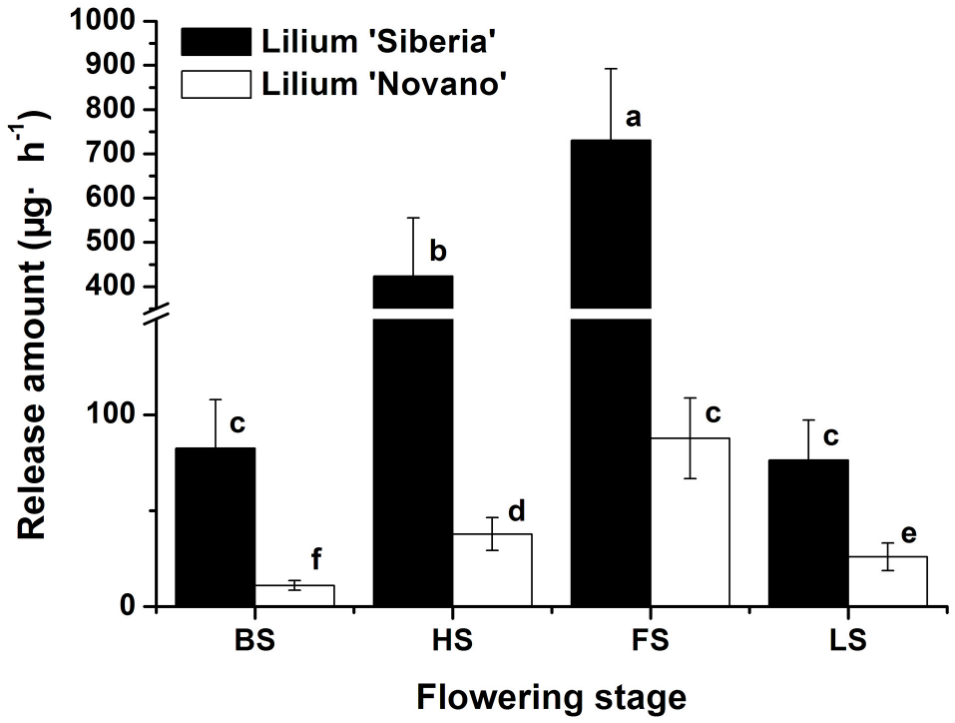
The total amount of floral scent emitted from *Lilium* ‘Siberia’ (Hu et al., 2016) and *Lilium* ‘Novano’. Each bar is the average of three independent biological replications, and standard errors are shown. Statistical significance [least significant difference (LSD)] of the difference in the amount of floral scent released is indicated by different small letters (*P* < 0.05).

Figure 1 showed the total amounts of floral scent emitted from *Lilium* ‘Siberia’ (Hu et al., 2016) and *Lilium* ‘Novano’ at different flowering stages. These values initially increased and then decreased with flower development, showing similar patterns. The maximum amounts released from *Lilium* ‘Siberia’ and *Lilium* ‘Novano’ occurred at FS, which were 7.85- and 6.90-fold higher, respectively, than those at BS. However, the amount released from *Lilium* ‘Siberia’ was significantly higher than that from *Lilium* ‘Novano’ (*P* < 0.05). At HS and FS in particular, the amounts released from *Lilium* ‘Siberia’ were ~11.19- and 8.32-fold higher, respectively, compared with those from *Lilium* ‘Novano’.

**Figure 2:**
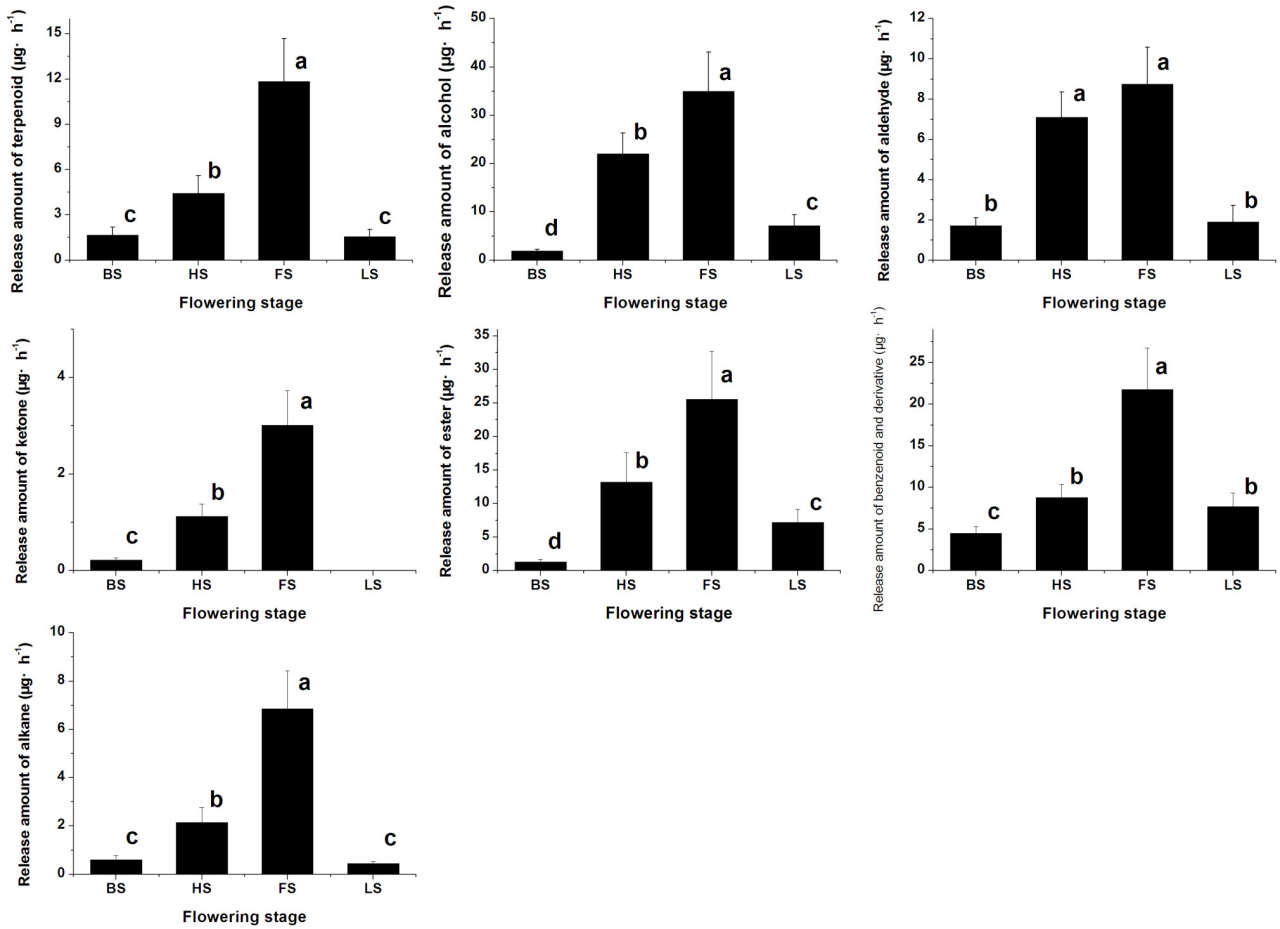
The amounts of different categories of components in the floral scent emitted by *Lilium* ‘Novano’. Each bar is the average of three independent biological replications, and standard errors are shown. Statistical significance [least significant difference (LSD)] of the difference in the amount of floral scent released is indicated by different small letters (*P* < 0.05).

We have detected 7 main categories of volatile components in the floral scents of *Lilium* ‘Siberia’ (Hu et al., 2016), including terpenoids, benzenoids and derivatives, alkanes, alcohols, aldehydes, ketones, and esters, which were also found in the floral scent of *Lilium* ‘Novano’ (Figure 2). The release amounts of these components also increased initially and then decreased during the flowering stage. The maximum release amount occurred at FS, showing a pattern similar to the total release amount. Among the volatile components of *Lilium* ‘Siberia’, the terpenoid compound showed the highest release amount (Hu et al., 2016). The release amounts of terpenoid compounds at different flowering stages accounted for over 72% of the total release amount from *Lilium* ‘Siberia’ (Table 1), but in *Lilium* ‘Novano’, the relative release amounts of terpenoid compounds did not exceed 16% (Table 1).

**Table 1:** The relative release amounts of volatile components of *Lilium* ‘Siberia’ and *Lilium* ‘Novano’.

***Lilium***	**Volatile components**	**Relative release amounts (%)**			
		**BS**	**HS**	**FS**	**LS**
*Lilium* ‘Siberia’	Terpenoid	78.91 ± 24.10	78.11 ± 30.17	80.22 ± 20.46	72.83 ± 22.05
	Benzenoid and derivative	5.96 ± 1.98	2.57 ± 0.94	3.60 ± 0.98	8.56 ± 2.46
	Alkane	–	1.31 ± 0.29	0.76 ± 0.17	–
	Alcohol	3.22 ± 1.00	8.07 ± 1.71	7.29 ± 1.56	7.54 ± 1.41
	Aldehyde	7.78 ± 2.22	2.67 ± 0.54	1.70 ± 0.31	3.24 ± 0.67
	Ketone	–	0.44 ± 0.07	0.30 ± 0.06	–
	Ester	4.12 ± 1.32	6.83 ± 2.02	6.14 ± 1.36	7.84 ± 1.26
*Lilium* ‘Novano’	Terpenoid	15.03 ± 4.68	11.70 ± 3.14	13.47 ± 3.26	6.04 ± 1.73
	Benzenoid and derivative	40.50 ± 7.29	23.22 ± 4.17	24.78 ± 5.71	29.64 ± 6.35
	Alkane	5.40 ± 1.62	5.65 ± 1.66	7.81 ± 1.79	1.73 ± 0.38
	Alcohol	17.10 ± 2.79	58.31 ± 11.18	39.87 ± 9.18	27.41 ± 8.78
	Aldehyde	15.39 ± 3.51	18.76 ± 3.30	9.98 ± 2.07	7.35 ± 3.16
	Ketone	1.98 ± 0.45	2.96 ± 0.69	3.43 ± 0.82	–
	Ester	11.79 ± 3.51	34.87 ± 11.57	29.14 ± 8.16	27.83 ± 7.39

*The relative release amounts indicate the percentages of release amounts of different volatile groups in the total release amount. “–”indicates that the compounds can not detected in the floral scent*.

Except for alkane and ketone, the amounts of the 5 other categories of components released from *Lilium* ‘Siberia’ (Hu et al., 2016) were higher than those released from *Lilium* ‘Novano’ at different flowering stages. At BS, HS, FS, and LS, the amounts of terpenoid compounds released from *Lilium* ‘Siberia’ were ~7.44-, 11.19-, 8.33-, and 2.94-fold higher, respectively, compared with *Lilium* ‘Novano’. Compared to *Lilium* ‘Novano’ (Figure 2), *Lilium* ‘Siberia’ emitted higher amounts of aldehydes (Hu et al., 2016) at BS and HS. The amounts of esters released from *Lilium* ‘Siberia’ (Hu et al., 2016) were also higher than those from *Lilium* ‘Novano’ at BS, HS, and FS, i.e., 2.60-, 2.19-, and 1.75-fold, respectively (Figure 2). In the floral scent of *Lilium* ‘Novano’, benzenoid and derivative and ester accounted for the high relative release amounts. So the analysis on release amounts of different components indicated that the significant differences in the amounts of the terpenoid compounds between *Lilium* ‘Siberia’ and *Lilium* ‘Novano’ mainly contributed to the difference in floral scent.

**Figure 3:**
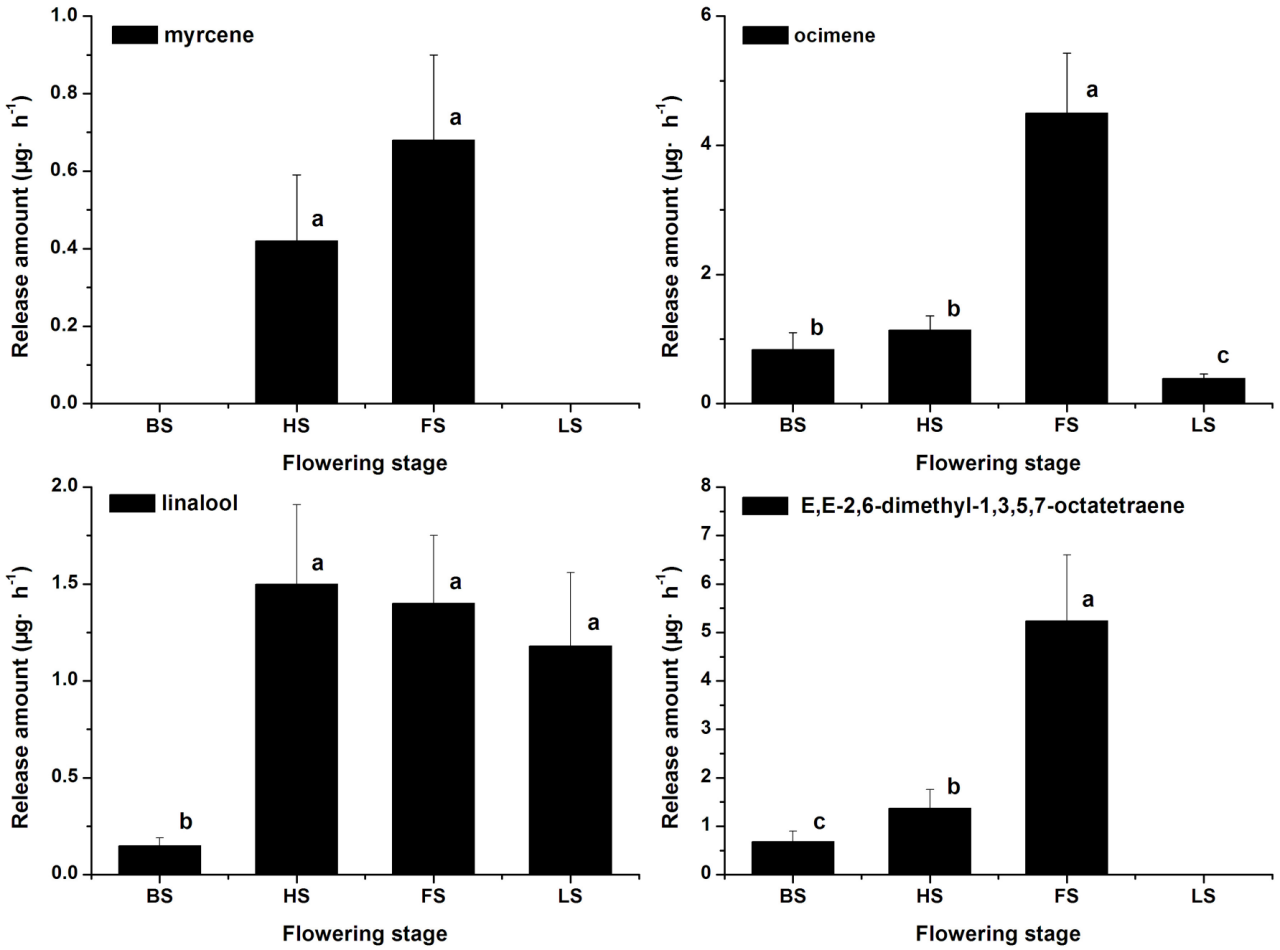
The amounts of terpenoid components emitted from *Lilium* ‘Novano’. Each bar is the average of three independent biological replications, and standard errors are shown. Statistical significance [least significant difference (LSD)] of the difference in the amounts of floral scent released is indicated by different small letters (*P* < 0.05).

The terpenoid components were also analyzed, and monoterpenes were found to be the dominant components in *Lilium* ‘Siberia’ (Hu et al., 2016). A total of 7 monoterpenes were detected in the floral scent of *Lilium* ‘Siberia’, including α-pinene, myrcene, ocimene, linalool, limonene, E,E-2,6-dimethyl-1,3,5,7-octatetraene, and 2,6-dimethyl-3,7-octadiene-2,6-diol (Hu et al., 2016). We found that the release amounts of the 7 monoterpenes all peaked at FS (Hu et al., 2016). In the floral scent of *Lilium* ‘Novano’ 4 monoterpenes, including myrcene, ocimene, linalool, and E,E-2,6-dimethyl-1,3,5,7-octatetraene, were found (Figure 3). Among these 4 compounds, E,E-2,6-dimethyl-1,3,5,7-octatetraene and ocimene were released in the highest amounts, but the maximum values did not exceed 6.00 μg·h^−1^ at FS. The release amount of myrcene was the lowest among the 4 monoterpenes, which only peaked at 0.68 ± 0.22 μg·h^−1^.

### High-throughput transcriptome sequencing and *de novo* assembly

To understand the molecular basis of the difference in the floral scent between *Lilium* ‘Siberia’ and *Lilium* ‘Novano’, the flowers at different developmental stages were used to build 6 libraries for high-throughput sequencing. We obtained a total of 29.24 Gb of raw data for the 6 samples (Table 2). We discarded low-quality reads, which contained adapters and unknown or low-quality bases, and after stringent quality checks and data cleaning, a total of 116.07 Mb of clean reads were obtained (Table 2). The GC (guanine + cytosine) contents of these samples were 47.26–50.23%, with an average of 49.27%. The average Q20 and Q3 percentage reached 95.46% and 90.59%, respectively (Table 2).

**Table 2:** Summary of sequencing and assembly data.

***Lilium***	**Sample ID**	**Clean base**	**Clean read**	**GC (%)**	**Q20 (%)**	**Q30 (%)**
*Lilium* ‘Siberia’	FS1	5,372,571,623	21,324,755	50.29	97.40	91.15
	FS2	4,603,927,794	18,275,930	49.77	97.71	91.74
	FS3	4,558,410,872	18,094,710	50.00	97.65	91.75
*Lilium* ‘Novano’	FS1	4,834,438,248	19,189,314	49.77	94.09	89.44
	FS2	4,692,162,610	18,624,569	50.82	93.86	89.07
	FS3	5,176,958,898	20,556,133	50.72	94.10	89.48
Average		4,873,078,341	19,344,235	50.23	95.80	90.44
Total		29,238,470,045	116,065,411	–	–	–

Based on the high quality reads, 2,864,438 contigs were assembled with an N50 length of 121 bp and an average length of 81 bp, including 94.67% contigs smaller than 200 bp. Using paired-end joining and gap-filling, the contigs were further assembled into 229,128 scaffolds with an N50 length of 1,472 bp and an average length of 876 bp, including 20,354 scaffolds larger than 2,000 bp. The *de novo* assembly yielded 124,233 unigenes with an N50 length of 986 bp and an average length of 615 bp (Table 3). Of these unigenes, 84.26% (104,682) were shorter than 1,000 bp, 10.48% (13,201) ranged from 1,000 to 2,000 bp, and the remaining 5.26% (6,530) were longer than 2,000 bp (Table 3). Among the unigenes longer than 2,000 bp, there were 2,212 unigenes whose lengths exceeded 3,000 bp, accounting for 1.78% of all unigenes.

**Table 3:** Length distribution of assembled contigs, scaffolds, and unigenes.

**Length range**	**Contig**	**Transcript**	**Unigene**
0–200	2,711,612(94.67%)	0	0
200–300	68,379 (2.39%)	66,077 (28.84%)	54,401 (43.79%)
300–500	37,889 (1.32%)	45,345 (19.79%)	29,518 (23.76%)
500–1000	25,884 (0.90%)	49,426 (21.57%)	20,763 (16.71%)
1000–2000	14,483 (0.51%)	45,226 (19.74%)	13,021 (10.48%)
2000+	6,191 (0.22%)	23,054 (10.06%)	6,530 (5.26%)
Total number	2,864,438	229,128	124,233
Total length	232,752,868	200,723,345	76,434,037
N50 length	121	1,472	986
Mean length	81	876	615

### Gene annotation and functional classification

All unigenes were aligned to 7 protein databases including COG, GO, KEGG, KOG, Swiss-Prot, and Nr using BLASTx with an *E*-value threshold of 10^−5^ and Pfam using HMMER with an *E*-value threshold of 10^−10^. As shown in Table 4, of 35,749 unigenes annotated, 34,717 (97.11%) unigenes presented significant BLASTx matches in the Nr database. There were 14,642 unigenes whose lengths were longer than 1000 bp, accounting for ~42.18% of 34,717 unigenes. Based on comparison against the Swiss-Prot database, 23,338 (65.28%) unigenes had significant matches, and ~49.65% (11,588) of unigenes were longer than 1,000 bp. In the Pfam and GO databases, 18,726 (52.38%) and 17,644 (49.36%) unigenes were also found to have significant matches respectively, and 6,210 (17.37%) unigenes were similar to proteins in the KEGG database.

**Table 4:** Statistics of annotation analysis of unigenes.

**Annotated databases**	**Unigene**	**Percentage (%)**	**≥300 nt**	**≥1,000 nt**
COG	9,183	25.69	7,878	5,620
GO	17,644	49.36	14,295	8,382
KEGG	6,210	17.37	5,292	3,376
KOG	10,557	29.53	15,741	9,150
Swiss-Prot	23,338	65.28	19,764	11,588
Nr	34,717	97.11	27,640	14,642
Pfam	18,726	52.38	16,711	11,517
All	35,749	100	28,050	14,715

**Figure 4:**
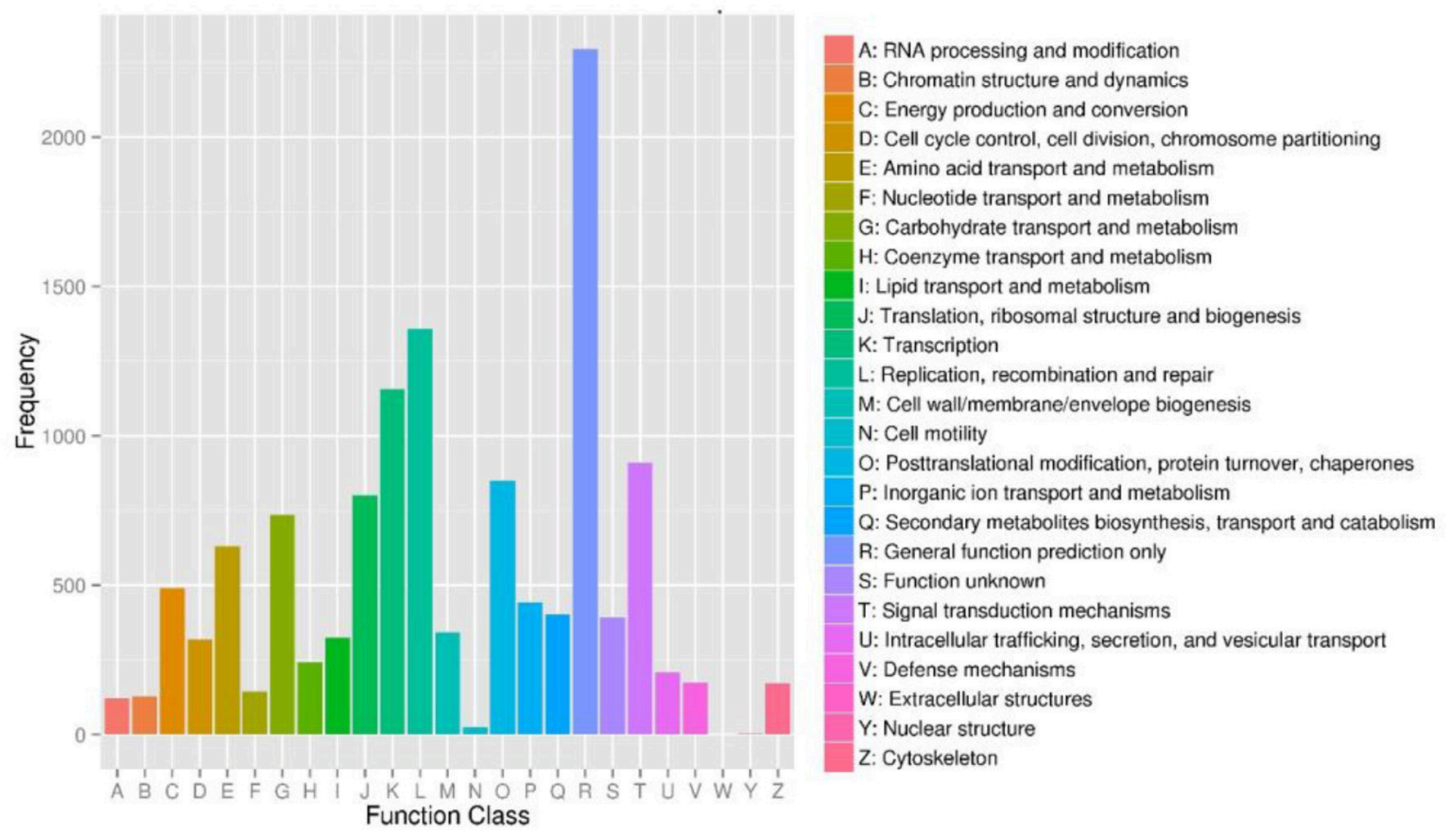
Cluster of orthologous groups (COG) functional classification of unigenes of *Lilium* ‘Siberia’ petals. From a total of 35,749 final unigenes, 9,183 annotated unigenes with significant homology in the COG database (*E*−value ≤ 1.0 E^−5^) were classified into 25 KOG categories.

To further evaluate the completeness of our transcriptome library and the effectiveness of our annotation process, we searched the annotated sequences for genes with COG (cluster of orthologous groups) classifications, and 9,183 unigenes were assigned to the COG classification (Figure 4). Among the 25 COG categories, the cluster for “General function prediction only” (2,295, 24.99%) represented the largest group, followed by “Replication, recombination and repair” (1,359, 14.80%), “Transcription” (1,155, 12.58%), and “Signal transduction mechanisms” (910, 7.19%). The “Secondary metabolites biosynthesis, transport and catabolism” category about which we were concerned accounted for 4.38% (402) of the sequences.

**Figure 5:**
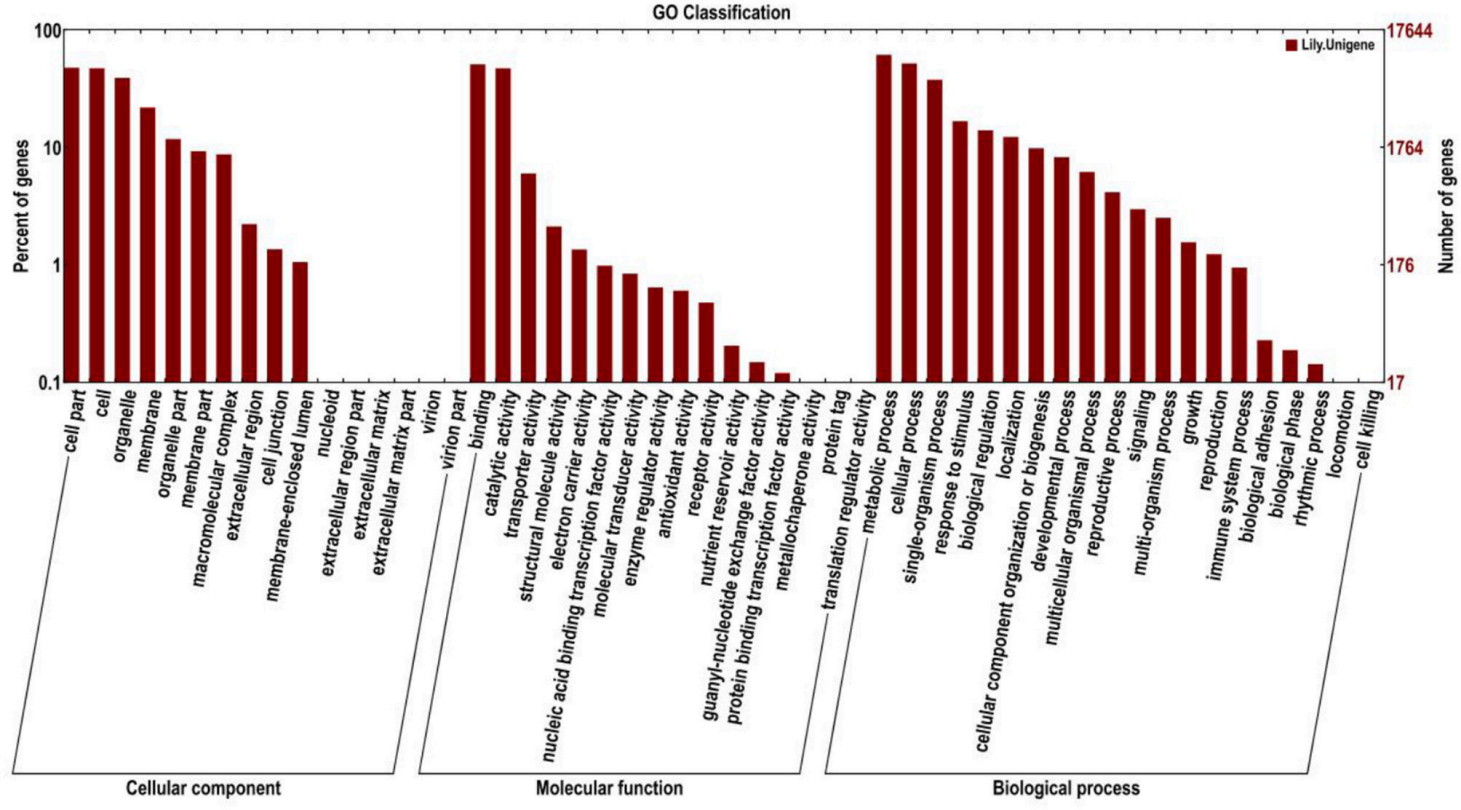
Histogram presentation of Gene Ontology (GO) classifications. Left y-axis indicates the percentage of unigenes in subcategories of each main category. Right y-axis indicates the number of unigenes in each subcategory.

Gene ontology (GO) was also used to classify the functions of the predicted unigenes. Based on the sequence homology, 17,644 sequences were categorized into 52 functional groups (Figure 5). The assigned functions of the unigenes covered a broad range of GO categories. The unigenes were assigned to three main categories including cellular component, molecular function, and biological process categories (Figure 5). Of these, the biological process category constituted the majority followed by the cellular component and molecular function categories. In the biological process category, the metabolic process (10,841, 61.44%) and cellular process (9,160, 51.92%) indicated that some important metabolic activities occurred in *Lilium* petals, including monoterpene biosynthesis. A total of 2,465 (13.97%) and 1456 (8.25%) unigenes were assigned to biological regulation and the developmental process, respectively. In the cellular component category, cell part (8,417, 47.70%), cell (8,343, 47.29%), and organelle (6,900, 39.11%) were prominently represented. In the molecular function category, binding (2,904, 16.46%) and catalytic activity (2,340, 13.26%) represented the majority. In addition, 1,057 (5.99%) unigenes were involved in transporter activity.

Based on the comparison against the KEGG database that records networks of molecular interactions and reactions, 6,210 unigenes were assigned to 118 KEGG pathways (Supplementary Table 2). These annotations provide a valuable resource for investigating the processes, functions, and pathways involved in floral scent biosynthesis.

In the comparison of floral scents between *Lilium* ‘Siberia’ and *Lilium* ‘Novano’, we found a significant difference in the amounts of monoterpenes released (Figure 3), which might be the key reason resulting in the fragrance difference. Thus, we were interested in the gene expression of the monoterpene biosynthesis. Genes involved in the pathways of terpenoid backbone biosynthesis and monoterpenoid biosynthesis are related to the production of monoterpenoid compounds in plants.

Through mapping to the KEGG reference pathways, a total of 39 annotated unigenes were assigned to the pathway of terpenoid backbone biosynthesis (Supplementary Table 3). Though no unigenes were mapped to the monoterpene biosynthesis pathway, based on GO function classification combined with the annotation of the Swissprot and nr databases, we found that 7 unigenes were annotated to monoterpene synthases mediating the biosynthesis of monoterpenes (Supplementary Table 4).

**Figure 6:**
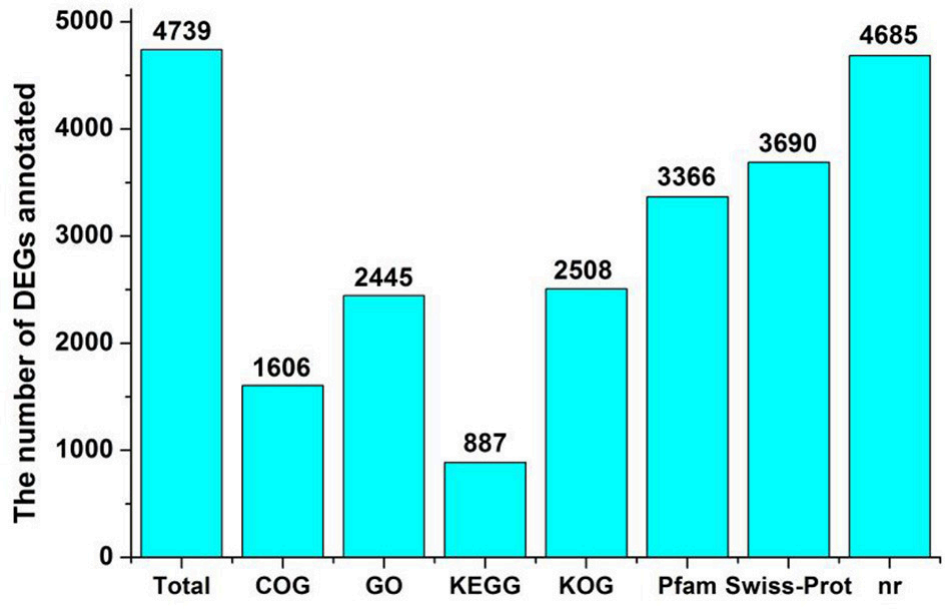
The number of DEGs annotated.

Differential gene expression of the two *Lilium* plants was analyzed at FS. We performed a statistical analysis on the genes with an RPKM value ≥2 to reduce false positives and false negatives. We filtered the data using an FDR ≤ 0.001 and |log2 (ratio)| ≥ 2. There was a significant difference in gene expression between the two *Lilium* plants in the heat-map (Supplementary Figure 1). The expression of 6,496 DEGs was found to be significantly changed between *Lilium* ‘Siberia’ and *Lilium* ‘Novano’, including 2,702 up-regulated unigenes and 3,794 down-regulated unigenes (NFS vs. SFS), among which 4,739 unigenes were annotated (Figure 6; Supplementary Table 5).

**Figure 7:**
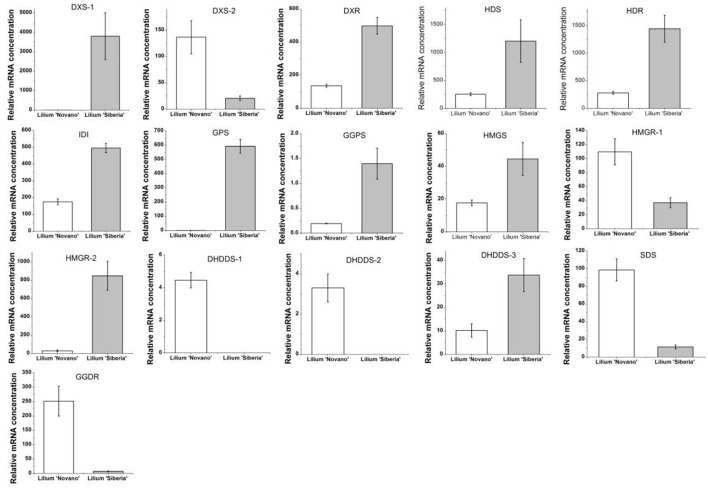
The expression profiles of 16 DEGs involved in the terpenoid backbone biosynthesis of *Lilium* ‘Siberia’ and *Lilium* ‘Novano’ based on RNA-Seq. The values represent the means of three independent biological replications, and standard errors are shown.

The enrichment factor of DEGs in the terpenoid backbone biosynthesis pathway was lower than in other pathways except sulfur metabolism, which indicated significant enrichment of DEGs in this pathway (Supplementary Figure 2). Among the DEGs annotated by the KEGG database, 16 unigenes were assigned to the terpenoid backbone biosynthesis (Table 5), and the sequence data were shown in Supplementary Table 6. Except *DXS-2*, these DEGs, including *DXS-1, DXR, HDS, HDR, IDI1, GPS*, and *GGPS*, in the MEP pathway upstream of monoterpene biosynthesis showed upregulated expression in *Lilium* ‘Siberia’ compared to *Lilium* ‘Novano’. The expression level of *DXS-1* in *Lilium* ‘Siberia’ was far higher (>6,000-fold) than that in *Lilium* ‘Novano’ (Figure 7). The expression level of *GPS* in *Lilium* ‘Siberia’ was 819-fold higher than that in *Lilium* ‘Novano’. In addition, the genes, *DHDDS-1, DHDDS-2, SDS*, and *GGDR*, which did not mediate monoterpenoid biosynthesis downstream of the MEP pathway presented downregulated expression in *Lilium* ‘Siberia’ (Table 5). In *Lilium* ‘Siberia’, the expression of *DHDDS-1* and *DHDDS-2* could not be detected (Figure 7). The gene expression of *HMGS* and *HMGR-2* in upstream of IPP biosynthesis was also upregulated in the MVA pathway (Table 5). The relative mRNA concentration of *HMGR-2* in *Lilium* ‘Siberia’ was nearly 31-fold higher than that in *Lilium* ‘Novano’ (Figure 7).

**Table 5:** The DEGs assigned to the terpenoid backbone biosynthesis.

**Sequence name**	**Enzyme/Gene**	**Regulated**
c64291.graph_c0	1-Deoxy-D-xylulose-5-phosphate synthase/*DXS-1*	up
c79428.graph_c0	1-Deoxy-D-xylulose-5-phosphate synthase/*DXS-2*	down
c76241.graph_c0	1-Deoxy-D-xylulose-5-phosphate reductoisomerase/*DXR*	up
c78346.graph_c0	4-Hydroxy-3-methylbut-2-enyl diphosphate synthase/*HDS*	up
c76487.graph_c0	4-Hydroxy-3-methylbut-2-enyl diphosphate reductase/*HDR*	up
c73716.graph_c0	Isopentenyl-diphosphate delta-isomerase/*IDI*	up
c62401.graph_c0	Geranyl diphosphate synthase/*GPS*	up
c31768.graph_c0	Geranylgeranyl pyrophosphate synthase/*GGPS*	up
c74470.graph_c1	3-Hydroxy-3-methylglutaryl-CoA synthase/*HMGS*	up
c75877.graph_c0	3-Hydroxy-3-methylglutaryl-CoA reductase/*HMGR-1*	down
c76279.graph_c1	3-Hydroxy-3-methylglutaryl-CoA reductase/*HMGR-2*	up
c46561.graph_c0	Dehydrodolichyl diphosphate synthase/*DHDDS-1*	down
c61651.graph_c0	Dehydrodolichyl diphosphate synthase/*DHDDS-2*	down
c65528.graph_c0	Dehydrodolichyl diphosphate synthase/*DHDDS-3*	up
c73826.graph_c0	Solanesyl-diphosphate synthase/*SDS*	down
c75625.graph_c0	Geranylgeranyl diphosphate reductase/*GGDR*	down
Total		16

**Figure 8:**
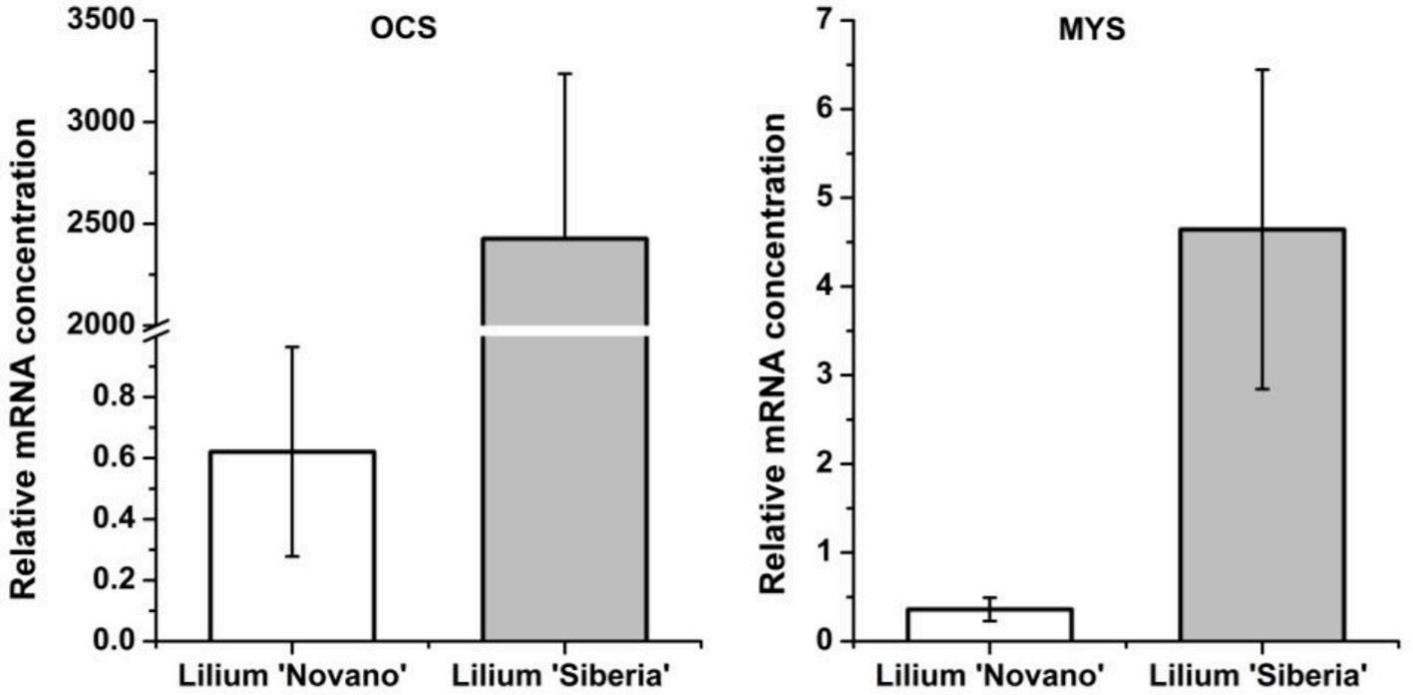
The expression profiles of *OCS* and *MYS* from *Lilium* ‘Siberia’ and *Lilium* ‘Novano’ based on RNA-Seq. The values indicate the means of three independent biological replications, and standard errors are shown.

Among the 7 DEGs annotated to monoterpene synthases, two unigenes annotated to the ocimene synthase gene (*OCS*, c65063.graph_c0) and myrcene synthase gene (*MYS*, c66796.graph_c0) respectively were upregulated in *Lilium* ‘Siberia’ (Figure 8). The relative mRNA concentration of the *OCS* in *Lilium* ‘Siberia’ almost reached 2,500, which was far higher than that in *Lilium* ‘Novano’. The relative mRNA concentration of the *OCS* in *Lilium* ‘Novano’ did not exceed 1. The expression level of *MYS* showed a similar pattern, which was nearly 12.91-fold in *Lilium* ‘Siberia’ higher than in *Lilium* ‘Novano’.

### Confirmation of differential gene expression by QRT-PCR

**Figure 9:**
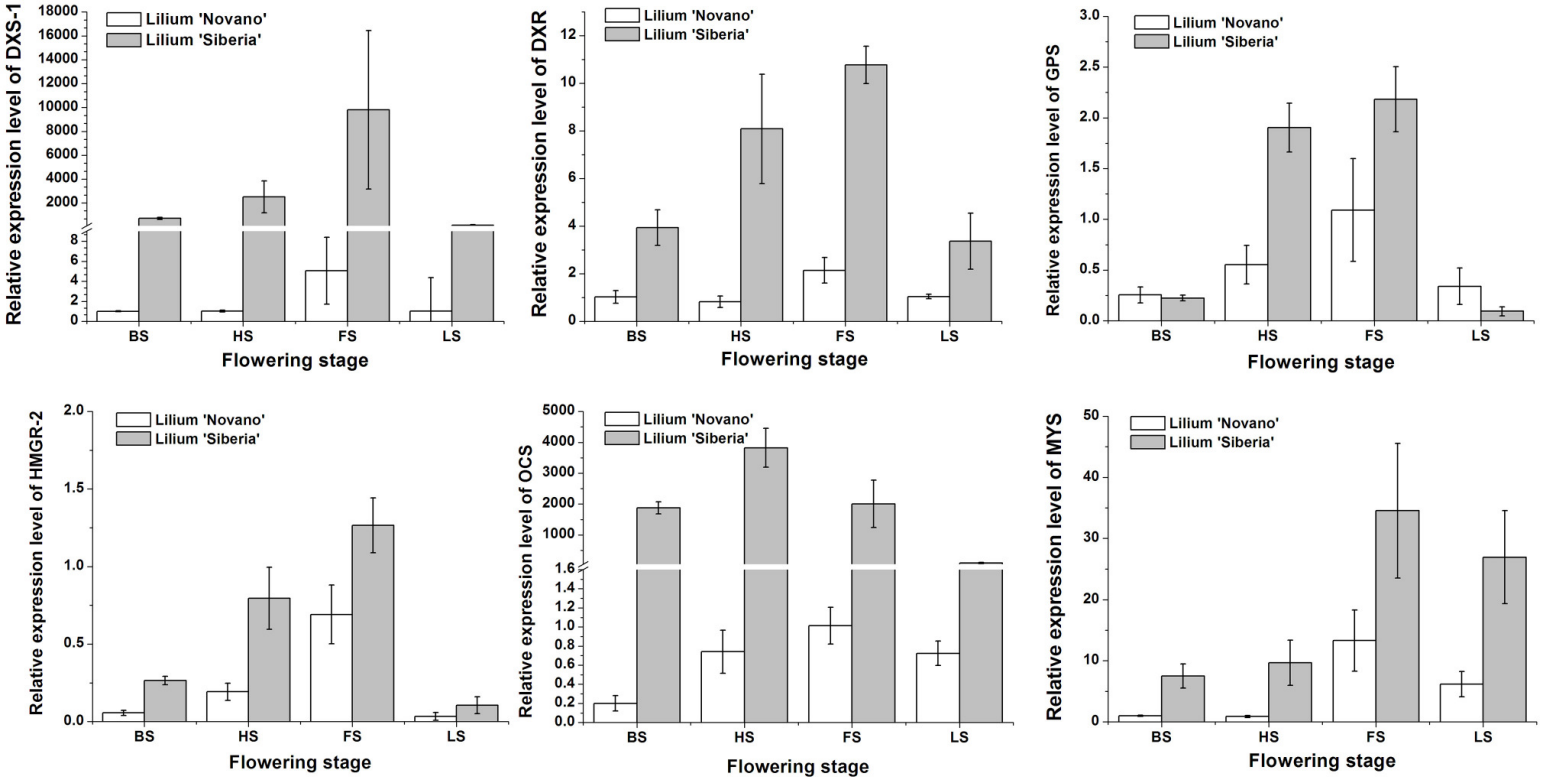
The relative expressions of *DXS-1, DXR, GPS, HMGR-2, OCS*, and *MYS* of *Lilium* ‘Siberia’ and *Lilium* ‘Novano’ at different flowering stages based on qRT-PCR. The values represent the means of three independent biological replications, and standard errors are shown.

Seven DEGs involved in monoterpene biosynthesis were selected for qRT-PCR analysis to validate the expression profiles obtained by RNA-Seq. The expression patterns of *DXS-1, DXR, GPS, HMGR-2, OCS*, and *MYS* of *Lilium* ‘Siberia’ and *Lilium* ‘Novano’ at the different flowering stages were shown in Figure 9. The expression of these genes exhibited a similar pattern in both genotypes, i.e., an initial increase and then a decrease with flower development (Figure 9). High expression levels were measured at HS and FS, which was consistent with the amounts of monoterpenes released (Figure 3).

Additionally, it was found that the relative expression levels of these 6 genes in *Lilium* ‘Siberia’ were higher than those in *Lilium* ‘Novano’ at the 4 stages, especially *DXS-1* and *OCS*, whose relative expression levels in *Lilium* ‘Siberia’ at FS were 9526- and 2015-fold higher, respectively, than those in *Lilium* ‘Novano’. This result was basically consistent with the RNA-Seq results.

## Discussion

Floral scent plays an important role in plant ecophysiology and the evaluation of ornamental quality and has become a new research focus in plant science. The components and release patterns of floral scent have been determined for many plants, but research is urgently needed to reveal the mechanisms of biosynthesis and regulation. As an important ornamental flower, *Lilium* has been cultivated for a long time, and hundreds of genotypes are bred. With respect to fragrance, *Lilium* genotypes range from unscented types to strongly scented types. However, the mechanism underlying fragrance differences among different *Lilium* genotypes is largely unknown. In this study, we investigated the differences in the components and amounts of floral scent between scented *Lilium* ‘Siberia’ and unscented *Lilium* ‘Novano’ and explored the molecular mechanism based on the RNA-Seq technique.

The total release amounts of floral scent showed a regular pattern, i.e., an initial increase followed by a decrease with flower development, in these two *Lilium* genotypes. In addition, a significant difference in the total release amounts was also found, with far higher amount released from *Lilium* ‘Siberia’ than from *Lilium* ‘Novano’. Moreover, analysis of the components showed that monoterpenes played a dominant role in the floral scent of *Lilium* ‘Siberia’, which were considered as the most important components. Therefore, the different emissions of monoterpenes mainly contributed to the difference in the floral scent between these two genotypes. Monoterpenes have been found to be the main components in the floral scent of numerous plant species. In our studies on the floral scents of tree peony (Zhao et al., 2012) and *Syringa* plants (Yang et al., 2016), different genotypes emitted different amounts of monoterpenes. A previous study showed that the high expression level of the terpene synthase gene was believed to be the main reason underlying the high amount of terpene released from scented *Alstroemeria* genotypes (Aros et al., 2012). Therefore, exploring the different expression of monoterpene biosynthesis-related genes is the breakthrough point for elucidating the molecular mechanism. The MVA and MEP pathways mediate the biosynthesis of monoterpenes. In addition to terpene synthase, the upstream enzymes of these pathways also play an important role in the regulation of monoterpene biosynthesis. Therefore, investigation of only terpene synthase gene expression is insufficient to disclose the molecular mechanism. The RNA-Seq technique is a powerful and attractive tool for in-depth analysis of multiple genes at the molecular level, especially for plants without genomic data, including *Lilium*.

In our study, we performed *de novo* sequence assembly of the flowers of *Lilium* ‘Siberia’ and *Lilium* ‘Novano’ at the full-bloom stage using RNA-Seq technique. Approximately 29.24 Gb of raw data were obtained and assembled into 124,233 unigenes, and 35,749 unigenes were annotated in the COG, GO, KEGG, KOG, Swiss-Prot, Nr, and Pfam databases. Through comparison of gene expression between these two *Lilium* genotypes, 6,496 DEGs were revealed. The DEGs were enriched in 20 pathways involved in photosynthesis, plant resistance, hormone signaling, and secondary metabolite synthesis, etc. According to the floral scent analysis, we focused on the pathways contributing to monoterpene biosynthesis.

Two metabolic pathways, i.e., the MVA and MEP pathways, are involved in monoterpene biosynthesis, among which the MEP pathway is the main pathway to produce monoterpenes. In the MEP pathway 10 enzymes including DXS, DXR, etc. mediate the biosynthesis of monoterpenes. Through DEG analysis of transcriptome data, we found that DEGs in the terpenoid backbone biosynthesis pathway containing MVA and MEP pathways were significantly enriched. In the MEP pathway, the gene expression of *DXS-1, DXR, HDS, HDR, IDI1, GPS*, and *GGPS* was upregulated in *Lilium* ‘Siberia’ compared to *Lilium* ‘Novano’, which indicated that the activation level of the MEP pathway in *Lilium* ‘Siberia’ was higher than in *Lilium* ‘Novano’, resulting in the accumulation of GPP, the precursor for the biosynthesis of monoterpenes.

DXS is determined to catalyze a rate-limiting step in the MEP pathway in plants using the transgenic *Arabidopsis* plants that over- or underexpress this enzyme (Estévez et al., 2001). Compared with non-transgenic wild-type plants, the transgenic plants accumulate different levels of various terpenoids, therefore, DXS acts as a limiting enzyme to control the biosynthesis of terpenoids in plants (Estévez et al., 2001). In our study, two DXS genes were differentially expressed between these two *Lilium* genotypes. *DXS-1* was upregulated and *DXS-2* was downregulated in *Lilium* ‘Siberia’ compared to *Lilium* ‘Novano’. However, the upregulated level of *DXS-1* was 750-fold higher than the downregulated level of *DXS-2*, thus, the integrated gene expression level of *DXS* in *Lilium* ‘Siberia’ was far higher than that in *Lilium* ‘Novano’. In the following experiment to confirm the gene expression, *DXS-1* showed a higher expression level at the 4 flowering stages in *Lilium* ‘Siberia’ than in *Lilium* ‘Novano’. Therefore, ample substrate was produced for the following enzyme, DXR, to drive the MEP pathway.

DXR catalyzes the second step of the MEP pathway. Inhibition of DXR activity by fosmidomycin leads to the depression of plastidial terpene biosynthesis (Rodríguez-Concepción et al., 2001; Huang et al., 2010). Overexpression of *DXR* in transgenic tobacco increases DXR activity, photosynthetic pigment content and volatile isoprenoid components (Yang et al., 2012). These studies demonstrate that DXR also plays an important role in the MEP pathway. Similar to *DXS-1*, a higher expression level of *DXR* was also found in the petals of *Lilium* ‘Siberia’ compared to *Lilium* ‘Novano’, especially at FS.

HDR, HDS, and IDI also play a key role in the regulation of isoprenoid biosynthesis in the MEP pathway. By applying [^13^C]DXP and [^14^C]DXP to the leaves of *Nicotiana benthamiana*, whose HDS and IDI genes were silenced by the tobacco rattle virus, Page et al. (2004) found that the MEP pathway was depressed, demonstrating the participation of HDS and IDI. The *Arabidopsis* mutant that *hdr-1* is knocked out is albino lethal, and the *HDR* transgene-induced gene silencing lines are albino, pale green, or variegated (Hsieh and Hsieh, 2015), confirming that HDR is essential for the MEP pathway in plants. In *Lilium* ‘Siberia’, the HDR, HDS, and IDI genes showed higher transcript expression compared with *Lilium* ‘Novano’ during the development of flower.

GPS (GPPS) is an important regulatory component involved in balancing a recombinant monoterpene biosynthesis pathway (Zhou et al., 2015). GPS has been described in *Mentha piperita* (Burke et al., 1999), *Arabidopsis* (Bouvier et al., 2000) *Abies grandis* (Burke and Croteau, 2002), and *Phalaenopsis bellina* (Hsiao et al., 2008), and participates in the biosynthesis of monoterpenes by producing GPP. A higher amount of precursor accumulated for monoterpene biosynthesis in the petals of *Lilium* ‘Siberia’ through a higher expression level of GPS (GPPS) compared with *Lilium* ‘Novano’.

In addition, in the MVA pathway the gene expression of HMGS and HMGR, two rate-limiting enzymes, showed higher levels in *Lilium* ‘Siberia’ than in *Lilium* ‘Novano’. Though *HMGR-1* was downregulated in *Lilium* ‘Siberia’ compared to *Lilium* ‘Novano’, the level was far lower than the upregulated level of *HMGR-2* in *Lilium* ‘Siberia’. Due to the connection of the MVA and MEP pathways through IPP, the IPP produced in the MVA pathway also can be supplied to the MEP pathway to synthesize monoterpenes. In *Saccharomyces cerevisiae*, the overexpression of *HMGR* enhanced the production of plant monoterpenes (Rico et al., 2010). It is believed that in *Lilium* ‘Siberia’, the MVA pathway is also more active than in *Lilium* ‘Novano’ to supply more IPP for the MEP pathway.

**Figure 10:**
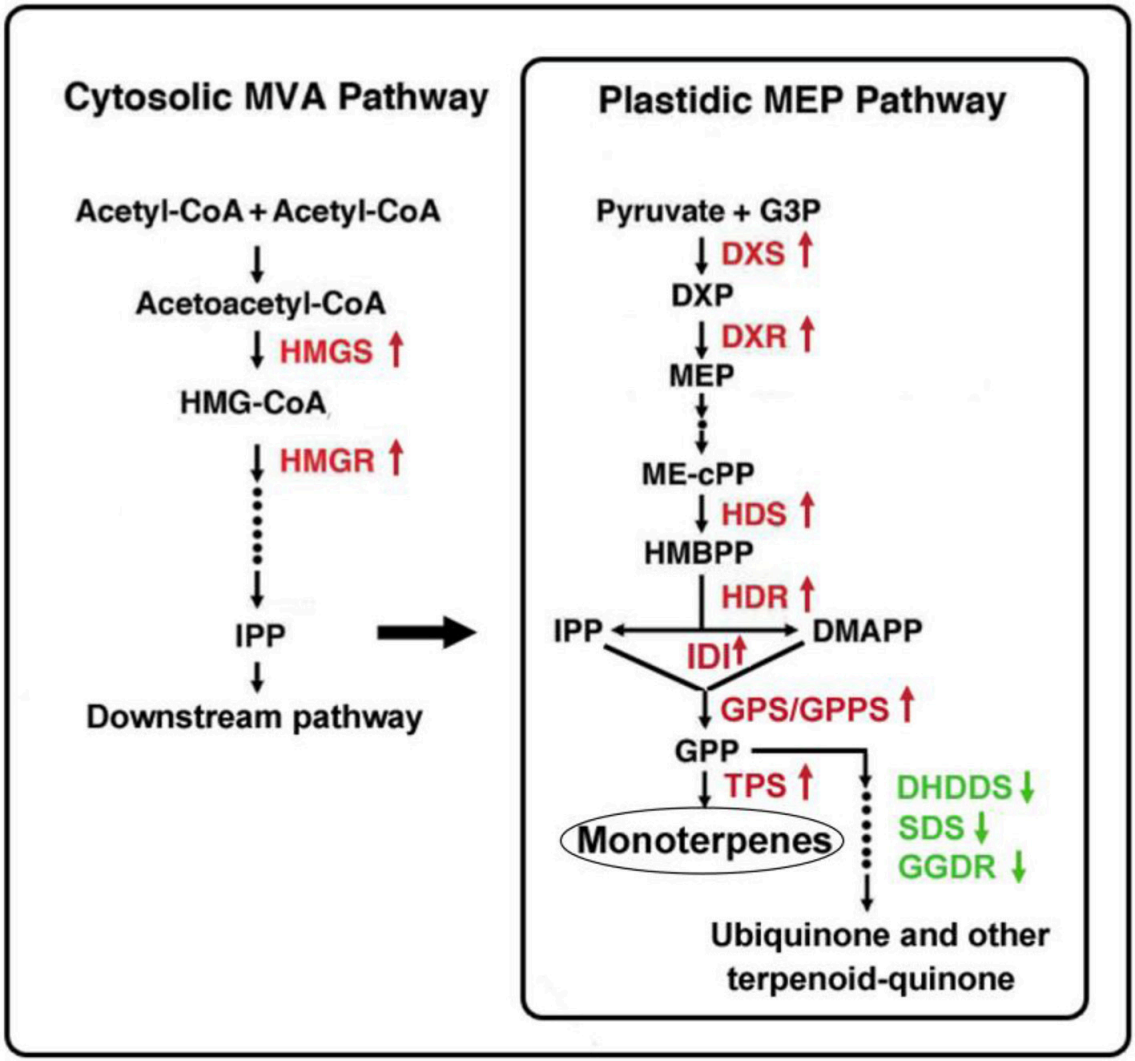
Gene expression regulation in monoterpene biosynthesis in the tepals of *Lilium* ‘Siberia’ compared to *Lilium* ‘Novano’. The red color indicates the upregulated genes, and the green color indicates the downregulated genes.

It is interesting that the downstream MEP pathway genes, DHDDS, SDS, and GGDR, which mediate the biosynthesis of ubiquinone and other terpenoid-quinones in the branched metabolic pathway, presented lower gene expression levels in *Lilium* ‘Siberia’ compared to *Lilium* ‘Novano’. In the final step of monoterpene biosynthesis, two monoterpene synthase genes, *OCS* and *MYS*, were expressed at high levels in the tepals of *Lilium* ‘Siberia’. This result indicated that in *Lilium* ‘Siberia’, the genes of the key enzymes in the MEP pathway were expressed at higher levels, resulting in the biosynthesis of more monoterpenes than *Lilium* ‘Novano’ (Figure 10). Moreover, the high activation level of the MVA pathway, together with the decrease in the branched metabolic pathway of ubiquinone and other terpenoid-quinones, may contribute to the high level of monoterpene biosynthesis (Figure 10).

Our study showed that the differential expression of genes involved in the monoterpene biosynthesis pathway led to differences in the amounts of monoterpenes between *Lilium* ‘Siberia’ and *Lilium* ‘Novano’. However, why the pathway had a high activation level in *Lilium* ‘Siberia’ remains unknown, which need to be investigated in the following study.

## Author contributions

ZH designed the experiments, analyzed the results, and wrote the manuscript. BT and QW performed the experiments. PL provided the idea, supervised the research work. JZ and KZ gave advice and guidance for the experiment.

### Conflict of interest statement

The authors declare that the research was conducted in the absence of any commercial or financial relationships that could be construed as a potential conflict of interest.

## Supplementary material

Supplementary Figure 1 The heat-map of the total differentially expressed genes (DEGs).

Supplementary Figure 2 The DEGs enrichment in different KEGG pathways.

Supplementary Table 1 The primers of unigenes.

Supplementary Table 2 KEGG pathways.

Supplementary Table 3 The unigenes assigned to the pathway of terpenoid backbone biosynthesis.

Supplementary Table 4 The unigenes assigned to the pathway of monoterpene biosynthesis.

Supplementary Table 5 The DEGs (NFS vs SFS).

Supplementary Table 6 Sequence information of DEGs.
