# Supplementary figures and images for: Transcriptome Sequencing Analysis Reveals a Difference in Monoterpene Biosynthesis between Scented Lilium ‘Siberia’ and Unscented Lilium ‘Novano’
Source: Front Plant Sci. 2017 Aug 4;8:1351. doi: 10.3389/fpls.2017.01351 (PMC5543080; doi:10.3389/fpls.2017.01351)

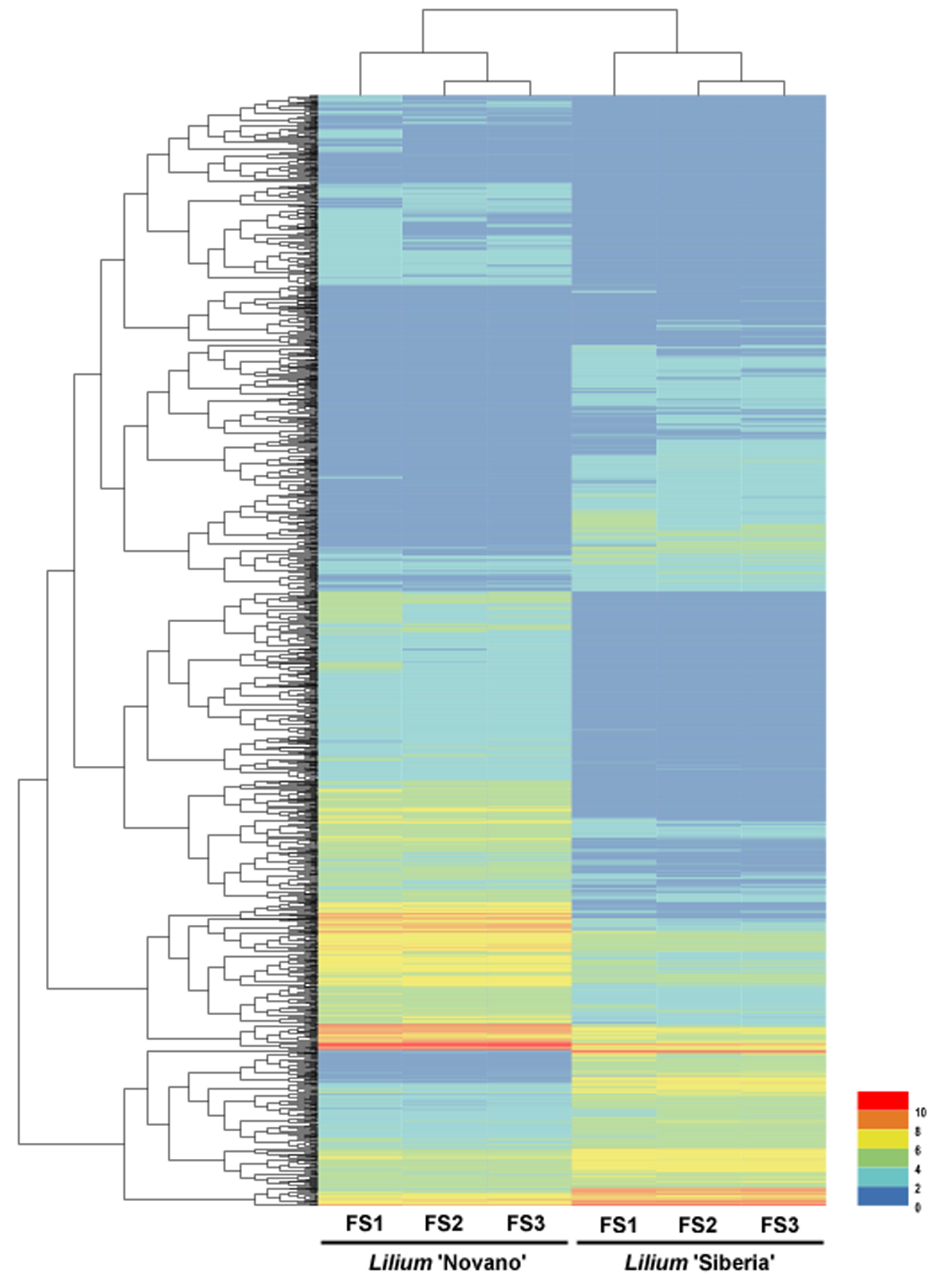

Supplement: Supplementary Figure 1 — The heat-map of the total differentially expressed genes (DEGs). [file Image1.JPEG]

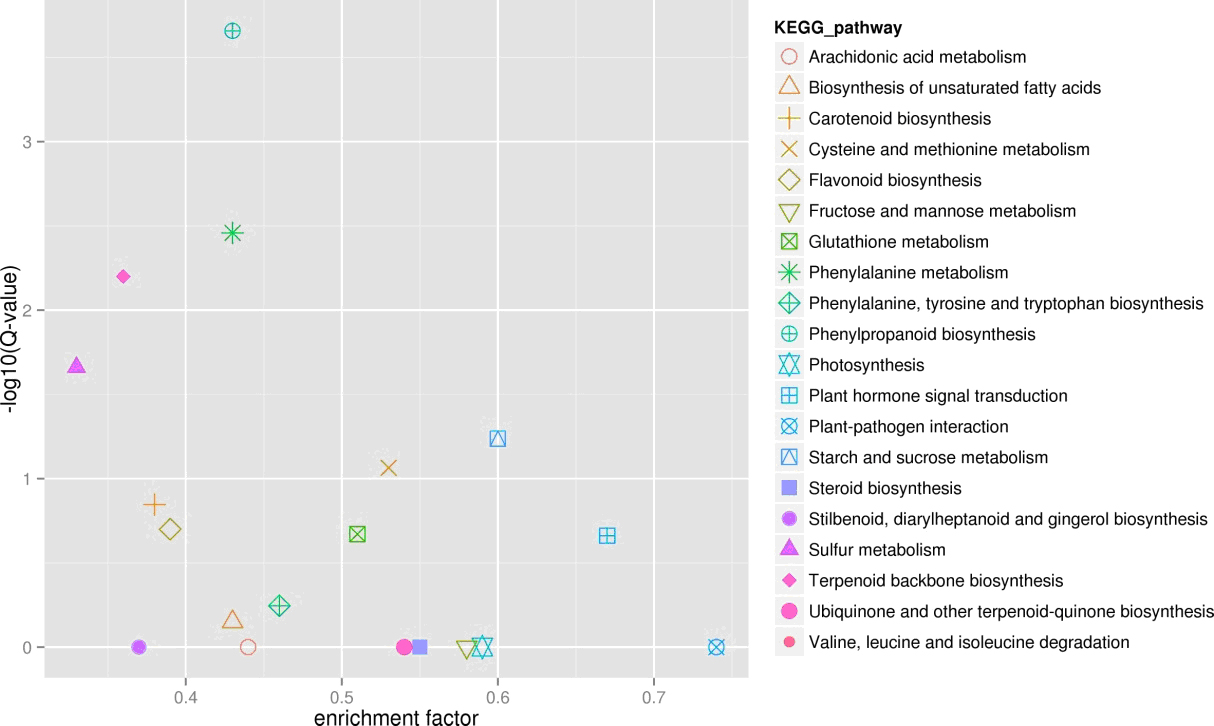

Supplement: Supplementary Figure 2 — The DEGs enrichment in different KEGG pathways. [file Image2.JPEG]
