# Supplementary material for: Transcriptome Sequencing Analysis Reveals a Difference in Monoterpene Biosynthesis between Scented Lilium ‘Siberia’ and Unscented Lilium ‘Novano’
Source: Front Plant Sci. 2017 Aug 4;8:1351. doi: 10.3389/fpls.2017.01351 (PMC5543080; doi:10.3389/fpls.2017.01351)
Supplement: Supplementary Table 1 — The primers of unigenes. [file Table1.DOC]

Table 1 The primers of unigenes

| **Primer** | **Sequence** |
| --- | --- |
| *DXS-1-F* | CGGATGAACACCATTAGGAAGA |
| *DXS-1-R* | CTAACTCAGATGCATGGCCTC |
| *DXR-F* | ATGGCAGCCCTGAAG |
| *DXR-R* | TCATACAGGAACTGGACTC |
| *GPS-F* | ATGTATTTCCGTCGGGCTCT |
| *GPS-R* | GGCTCACTTTGTTCTTGTGAT |
| *HMGR-2-F* | GCAATGGACCTCCGCC |
| *HMGR-2-R* | CCGCTAAGGAAATACGAATACC |
| *OCS-F* | ATGGCAGCTATGAGCTGTTTC |
| *OCS-R* | TCATTCCAATGGGACATTATTGA |
| *MYS-F* | CGGATGGCGTTCGCACT |
| *MYS-R* | GGCTTAAATGCGTTCGATAAACA |
| *β-Actin-F* | CGGTGTCTGGATTGGAGGGTCA |
| *β-Actin-R* | CTTCCTGTGGACGATGGCTGGA |
