# Supplementary material for: Transcriptome Sequencing Analysis Reveals a Difference in Monoterpene Biosynthesis between Scented Lilium ‘Siberia’ and Unscented Lilium ‘Novano’
Source: Front Plant Sci. 2017 Aug 4;8:1351. doi: 10.3389/fpls.2017.01351 (PMC5543080; doi:10.3389/fpls.2017.01351)
Supplement: Supplementary Table 2 — KEGG pathways. [file Table2.DOC]

Table 2 KEGG pathways

| **NO.** | **#pathway** | **pathway_id** |
| --- | --- | --- |
| 1 | Glycolysis / Gluconeogenesis | ko00010 |
| 2 | Citrate cycle (TCA cycle) | ko00020 |
| 3 | Pentose phosphate pathway | ko00030 |
| 4 | Pentose and glucuronate interconversions | ko00040 |
| 5 | Fructose and mannose metabolism | ko00051 |
| 6 | Galactose metabolism | ko00052 |
| 7 | Ascorbate and aldarate metabolism | ko00053 |
| 8 | Fatty acid biosynthesis | ko00061 |
| 9 | Fatty acid elongation in mitochondria | ko00062 |
| 10 | Fatty acid metabolism | ko00071 |
| 11 | Synthesis and degradation of ketone bodies | ko00072 |
| 12 | Steroid biosynthesis | ko00100 |
| 13 | Ubiquinone and other terpenoid-quinone biosynthesis | ko00130 |
| 14 | Oxidative phosphorylation | ko00190 |
| 15 | Photosynthesis | ko00195 |
| 16 | Photosynthesis - antenna proteins | ko00196 |
| 17 | Purine metabolism | ko00230 |
| 18 | Caffeine metabolism | ko00232 |
| 19 | Pyrimidine metabolism | ko00240 |
| 20 | Alanine, aspartate and glutamate metabolism | ko00250 |
| 21 | Glycine, serine and threonine metabolism | ko00260 |
| 22 | Cysteine and methionine metabolism | ko00270 |
| 23 | Valine, leucine and isoleucine degradation | ko00280 |
| 24 | Valine, leucine and isoleucine biosynthesis | ko00290 |
| 25 | Lysine biosynthesis | ko00300 |
| 26 | Lysine degradation | ko00310 |
| 27 | Arginine and proline metabolism | ko00330 |
| 28 | Histidine metabolism | ko00340 |
| 29 | Tyrosine metabolism | ko00350 |
| 30 | Phenylalanine metabolism | ko00360 |
| 31 | Tryptophan metabolism | ko00380 |
| 32 | Phenylalanine, tyrosine and tryptophan biosynthesis | ko00400 |
| 33 | beta-Alanine metabolism | ko00410 |
| 34 | Taurine and hypotaurine metabolism | ko00430 |
| 35 | Selenocompound metabolism | ko00450 |
| 36 | Cyanoamino acid metabolism | ko00460 |
| 37 | Glutathione metabolism | ko00480 |
| 38 | Starch and sucrose metabolism | ko00500 |
| 39 | N-Glycan biosynthesis | ko00510 |
| 40 | Other glycan degradation | ko00511 |
| 41 | Other types of O-glycan biosynthesis | ko00514 |
| 42 | Amino sugar and nucleotide sugar metabolism | ko00520 |
| 43 | Glycosaminoglycan degradation | ko00531 |
| 44 | Glycerolipid metabolism | ko00561 |
| 45 | Inositol phosphate metabolism | ko00562 |
| 46 | Glycosylphosphatidylinositol(GPI)-anchor biosynthesis | ko00563 |
| 47 | Glycerophospholipid metabolism | ko00564 |
| 48 | Ether lipid metabolism | ko00565 |
| 49 | Arachidonic acid metabolism | ko00590 |
| 50 | Linoleic acid metabolism | ko00591 |
| 51 | alpha-Linolenic acid metabolism | ko00592 |
| 52 | Sphingolipid metabolism | ko00600 |
| 53 | Glycosphingolipid biosynthesis - globo series | ko00603 |
| 54 | Glycosphingolipid biosynthesis - ganglio series | ko00604 |
| 55 | Pyruvate metabolism | ko00620 |
| 56 | Glyoxylate and dicarboxylate metabolism | ko00630 |
| 57 | Propanoate metabolism | ko00640 |
| 58 | Butanoate metabolism | ko00650 |
| 59 | C5-Branched dibasic acid metabolism | ko00660 |
| 60 | One carbon pool by folate | ko00670 |
| 61 | Carbon fixation in photosynthetic organisms | ko00710 |
| 62 | Thiamine metabolism | ko00730 |
| 63 | Riboflavin metabolism | ko00740 |
| 64 | Vitamin B6 metabolism | ko00750 |
| 65 | Nicotinate and nicotinamide metabolism | ko00760 |
| 66 | Pantothenate and CoA biosynthesis | ko00770 |
| 67 | Biotin metabolism | ko00780 |
| 68 | Lipoic acid metabolism | ko00785 |
| 69 | Folate biosynthesis | ko00790 |
| 70 | Porphyrin and chlorophyll metabolism | ko00860 |
| 71 | Terpenoid backbone biosynthesis | ko00900 |
| 72 | Limonene and pinene degradation | ko00903 |
| 73 | Diterpenoid biosynthesis | ko00904 |
| 74 | Brassinosteroid biosynthesis | ko00905 |
| 75 | Carotenoid biosynthesis | ko00906 |
| 76 | Zeatin biosynthesis | ko00908 |
| 77 | Nitrogen metabolism | ko00910 |
| 78 | Sulfur metabolism | ko00920 |
| 79 | Phenylpropanoid biosynthesis | ko00940 |
| 80 | Flavonoid biosynthesis | ko00941 |
| 81 | Anthocyanin biosynthesis | ko00942 |
| 82 | Flavone and flavonol biosynthesis | ko00944 |
| 83 | Stilbenoid, diarylheptanoid and gingerol biosynthesis | ko00945 |
| 84 | Isoquinoline alkaloid biosynthesis | ko00950 |
| 85 | Tropane, piperidine and pyridine alkaloid biosynthesis | ko00960 |
| 86 | Aminoacyl-tRNA biosynthesis | ko00970 |
| 87 | Biosynthesis of unsaturated fatty acids | ko01040 |
| 88 | ABC transporters | ko02010 |
| 89 | Ribosome biogenesis in eukaryotes | ko03008 |
| 90 | Ribosome | ko03010 |
| 91 | RNA transport | ko03013 |
| 92 | mRNA surveillance pathway | ko03015 |
| 93 | RNA degradation | ko03018 |
| 94 | RNA polymerase | ko03020 |
| 95 | Basal transcription factors | ko03022 |
| 96 | DNA replication | ko03030 |
| 97 | Spliceosome | ko03040 |
| 98 | Proteasome | ko03050 |
| 99 | Protein export | ko03060 |
| 100 | Base excision repair | ko03410 |
| 101 | Nucleotide excision repair | ko03420 |
| 102 | Mismatch repair | ko03430 |
| 103 | Homologous recombination | ko03440 |
| 104 | Non-homologous end-joining | ko03450 |
| 105 | Phosphatidylinositol signaling system | ko04070 |
| 106 | Plant hormone signal transduction | ko04075 |
| 107 | Ubiquitin mediated proteolysis | ko04120 |
| 108 | Sulfur relay system | ko04122 |
| 109 | SNARE interactions in vesicular transport | ko04130 |
| 110 | Regulation of autophagy | ko04140 |
| 111 | Protein processing in endoplasmic reticulum | ko04141 |
| 112 | Endocytosis | ko04144 |
| 113 | Phagosome | ko04145 |
| 114 | Peroxisome | ko04146 |
| 115 | Plant-pathogen interaction | ko04626 |
| 116 | Natural killer cell mediated cytotoxicity | ko04650 |
| 117 | Circadian rhythm - mammal | ko04710 |
| 118 | Circadian rhythm – plant | ko04712 |
