# Supplementary material for: Transcriptome Sequencing Analysis Reveals a Difference in Monoterpene Biosynthesis between Scented Lilium ‘Siberia’ and Unscented Lilium ‘Novano’
Source: Front Plant Sci. 2017 Aug 4;8:1351. doi: 10.3389/fpls.2017.01351 (PMC5543080; doi:10.3389/fpls.2017.01351)
Supplement: Supplementary Table 3 — The unigenes assigned to the pathway of terpenoid backbone biosynthesis. [file Table3.DOC]

Table 3 The unigenes assigned to the pathway of terpenoid backbone biosynthesis

| Pathway | Pathway_id | Gene_number | Gene_id |
| --- | --- | --- | --- |
| Terpenoid backbone biosynthesis | ko00900 | 39 | c111374.graph_c0;c137403.graph_c0;c23247.graph_c0;c25223.graph_c0;c31768.graph_c0;c45470.graph_c0;c46561.graph_c0;c50341.graph_c0;c61651.graph_c0;c62401.graph_c0;c63495.graph_c0;c64291.graph_c0;c65528.graph_c0;c66722.graph_c0;c72392.graph_c0;c72759.graph_c0;c73099.graph_c0;c73229.graph_c0;c73716.graph_c0;c73826.graph_c0;c73831.graph_c0;c73930.graph_c0;c74470.graph_c1;c74630.graph_c0;c75510.graph_c0;c75625.graph_c0;c75877.graph_c0;c76241.graph_c0;c76279.graph_c1;c76487.graph_c0;c76706.graph_c0;c78346.graph_c0;c78533.graph_c0;c78797.graph_c0;c79428.graph_c0;c80759.graph_c0;c91691.graph_c0;c97162.graph_c0;c97395.graph_c0; |
