# Supplementary material for: Transcriptome Sequencing Analysis Reveals a Difference in Monoterpene Biosynthesis between Scented Lilium ‘Siberia’ and Unscented Lilium ‘Novano’
Source: Front Plant Sci. 2017 Aug 4;8:1351. doi: 10.3389/fpls.2017.01351 (PMC5543080; doi:10.3389/fpls.2017.01351)
Supplement: Supplementary Table 4 — The unigenes assigned to the pathway of monoterpene biosynthesis. [file Table4.DOC]

Table 4 The unigenes assigned to the pathway of monoterpene biosynthesis

| Pathway | Pathway_id | Gene_number | Gene_id |
| --- | --- | --- | --- |
| Monoterpene biosynthesis | ko00902 | 7 | c11974.graph_c0;c66796.graph_c0; c50546.graph_c0;c45569.graph_c0; c47492.graph_c0;c21085.graph_c0; c65063.graph_c0 |

|  |
| --- |
|  |
|  |
|  |
|  |
|  |
